# Adrenergic regulation of innate immunity: a review

**DOI:** 10.3389/fphar.2015.00171

**Published:** 2015-08-13

**Authors:** Angela Scanzano, Marco Cosentino

**Affiliations:** Center for Research in Medical Pharmacology, University of InsubriaVarese, Italy

**Keywords:** noradrenaline, adrenaline, adrenoceptors, innate immunity, immunity of CNS

## Abstract

The sympathetic nervous system has a major role in the brain-immune cross-talk, but few information exist on the sympathoadrenergic regulation of innate immune system. The aim of this review is to summarize available knowledge regarding the sympathetic modulation of the innate immune response, providing a rational background for the possible repurposing of adrenergic drugs as immunomodulating agents. The cells of immune system express adrenoceptors (AR), which represent the target for noradrenaline and adrenaline. In human neutrophils, adrenaline and noradrenaline inhibit migration, CD11b/CD18 expression, and oxidative metabolism, possibly through β-AR, although the role of α_1_- and α_2_-AR requires further investigation. Natural Killer express β-AR, which are usually inhibitory. Monocytes express β-AR and their activation is usually antiinflammatory. On murine Dentritic cells (DC), β-AR mediate sympathetic influence on DC-T cells interactions. In human DC β_2_-AR may affect Th1/2 differentiation of CD4+ T cells. In microglia and in astrocytes, β_2_-AR dysregulation may contribute to neuroinflammation in autoimmune and neurodegenerative disease. In conclusion, extensive evidence supports a critical role for adrenergic mechanisms in the regulation of innate immunity, in peripheral tissues as well as in the CNS. Sympathoadrenergic pathways in the innate immune system may represent novel antiinflammatory and immunomodulating targets with significant therapeutic potential.

## Introduction

### Physiology and pharmacology of adrenergic pathways

**Figure 1:**
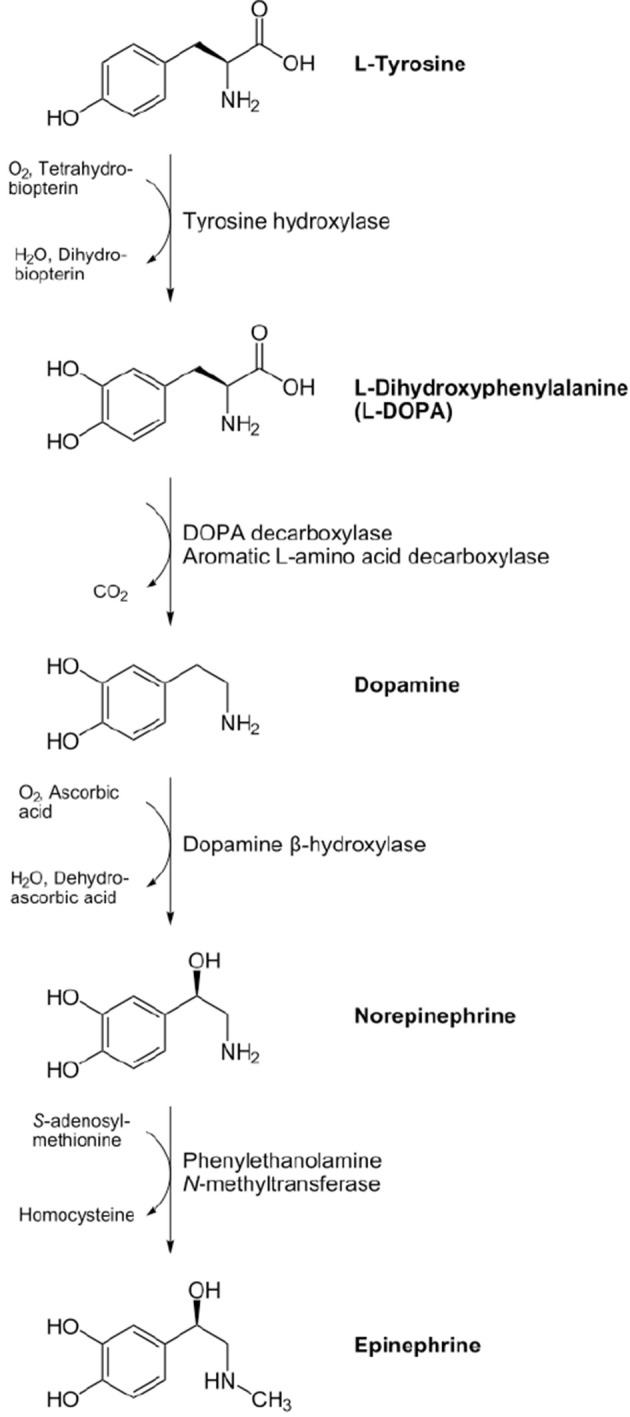
**Biosynthetic pathway of the catecholamines noradrenaline and adrenaline from the aminoacid tyrosine**. The synthesizing enzymes are shown to the right of each arrow, while enzyme cofactors are shown to the left (reproduced from the Wikimedia Commons—http://commons.wikimedia.org).

Adrenaline (“near the kidney,” from Latin roots *ad* and *renes*; US: epinephrine, from the Greek roots *epi* and *nephros*, i.e., “on the kidney”) belongs together with noradrenaline (the prefix “nor” standing for *nitrogen öhne radikal*, indicating the absence of a methyl group) to catecholamines, a group of chemicals containing a catechol or 3,4-dihydroxyphenyl group and an amine function. The first step in the synthesis of Noradrenaline is the transformation of the aminoacid tyrosine in Levodopa through the enzyme tyrosine hydroxilase that is the key rate-limiting enzyme in the biosynthetic pathway of Noradrenaline.Levodopa is decaborxlated into dopamine and finally noradrenaline is synthesized from dopamine by dopamine β-hydroxylase and is converted to adrenaline by phenylethanolamine N-methyltransferase (Figure 1). Adrenaline was isolated as pure crystalline base in 1900 by Jokichi Takamine in New Jersey and was the first hormone to be isolated in a pure state, while noradrenaline was proved in 1949 by Ulf von Euler in Stokholm to be the main sympathomimetic neurotransmitter in humans.

Noradrenaline act as neurotransmitter in the central and peripheral nervous systems. The sympathetic nervous system, through its preganglionic fibers, stimulates chromaffin cells in the adrenal glands to release into the bloodstream adrenaline (~80% in humans) and noradrenaline (~20%). In the brain, noradrenergic neurons are located mainly in the *locus coeruleus* (LC), and their axons project to hippocampus, septum, hypothalamus and thalamus, cortex and amygdala, to cerebellum, as well as to spinal cord. Brain adrenergic pathways control attention, arousal and vigilance, and regulate hunger and feeding behavior. Some central nervous system (CNS) neurons, mainly located in the medullary reticular formation, utilize adrenaline as the main neurotransmitter, possibly contributing to the modulation of eating behavior and to blood pressure regulation. In the periphery, noradrenaline is the main transmitter of sympathetic postganglionic fibers. Main direct effects of noradrenaline and adrenaline on peripheral tissues include: smooth muscle contraction in blood vessels supplying skin, kidney, and mucous membranes, stimulation of exocrine glands, smooth muscle relaxation in the gut wall, bronchi, and blood vessels supplying skeletal muscle, increases of heart rate and force of contraction, increased glycogenolysis in liver and muscle, lipolysis in adipose tissue, thermogenesis in the brown adipose tissue, modulation of the secretion of insulin and rennin (Feldman et al., 1997).

### Adrenergic receptors

The effects of noradrenaline and adrenaline are mediated by 7-transmembrane, G-protein coupled receptors called “adrenergic receptors” or “adrenoceptors” (AR) and classified in three major types—α_1_, α_2_, and β—each further divided into three subtypes, which are widely expressed throughout the CNS and in virtually all peripheral tissues (Ahlquist, 1948; Bylund et al., 1994).

The order of potency in the activation of these receptors by physiological ligands is noradrenaline > adrenaline for the α_1_- and α_2_-AR and adrenaline > noradrenaline for the β-AR.

Activation of α_1_-AR by adrenergic agonists induces the stimulation of a Gq and consequent phospholipase C (PLC) activation that promotes hydrolysis of phosphatidylinositol bisphosphate producing inositol trisphosphate and diacylglycerol. The results of this activation is the release of Ca^++^ as second messenger from non-mitochondrial pools or protein kinase C (PKC) and mediating intracellular release (Bylund et al., 1994). The α_2_-AR are considered inhibitory receptors; their activation induces the stimulation of a Gi resulting in adenylate cyclase inhibition and reduction of cyclic adenosine monophosphate (Bylund et al., 1994). Presynaptic autoreceptors mediating inhibition of neurotransmitter release are mainly α_2_-AR, while postsynaptic AR include all subtypes.

β-AR are coupled to a stimulatory Gs that leads to activation of adenylate cyclase and accumulation of the second messenger cAMP. In some situations, the β_3_-AR, can be coupled to Gi as well as to Gs (Gauthier et al., 1996). Receptors stimulation induces the protein kinase A (PKA) activation and phosporilation of L-type Ca^++^ channels and Ca^++^ entry (Guimarães and Moura, 2001). The β_1_-AR is the most important receptor that mediates cardiovascular responses to noradrenaline released from sympathetic nerve terminals and to circulating adrenaline. β_2_-AR are primarily localized on airway smooth muscle cells and are known to be involved in bronchial muscle relaxation.

AR ligands are used as drug therapeutics in different cardiovascular diseases such as hypertension, angina pectoris, congestive heart failure, or other diseases affecting million of individuals such as asthma, depression, benign prostatic hypertrophy, and glaucoma, as well as in shock, premature labor, opioid withdrawal, and as adjunct medications in general anesthesia (Bylund et al., 1994; Perez et al.) ^1^. Agonists of β_2_-AR are employed in therapy as first line-treatment of asthma and chronic obstructive pulmonary disease. The β_3_-AR is known to be located primarily on adipocytes (Harms et al., 1974), but at present no ligands of this receptors subtype are employed in therapy. The physiopharmacology of AR is summarized in Table 1.

**Table 1:** **Classification of AR (Perez et al.)**.

	**Main transduction mechanisms**	**Human tissue distribution**	**Physiological functions**	**Therapeutic drugs (indications)**
α_1A_	G_q_/G_11_ (phospholipase C stimulation, calcium channel)	Cerebral cortex, cerebellum, heart, liver, predominant subtype in prostate and urethra, lymphocytes	Contraction of urethral smooth muscle, contraction of skeletal muscle resistance arteries, contraction of human subcutaneous arteries	Agonists: methoxamine, methylnoradrenaline, midodrine, oxymetazoline, metaraminol, phenylephrine (vasoconstriction and mydriasis, used as vasopressors, nasal decongestants, and eye exams)
α_1B_		Spleen and kidney, somatic arteries and veins, endothelial cells, lymphocytes, osteoblasts	Contraction of arteries and veins, osteoblast proliferation	Antagonists: alfuzosin, doxazosin, phenoxybenzamine, phentolamine, prazosin, tamsulosin, terazosin, trazodone
α_1D_		Cerebral cortex, aorta, blood vessels of prostate, human bladder, lymphocytes	Contraction of arteries, ureteral contraction	(hypertension, benign prostatic hyperplasia)
α_2A_	G_i_/G_o_ (adenylate cyclase inhibition, potassium channel, calcium channel, phospholipase A2 stimulation)	Brain > spleen > kidney > aorta = lung = skeletal muscle > heart = liver	Presynaptic inhibition of noradrenaline release, hypotension, sedation, analgesia, hypothermia	Agonists: dexmedetomidine, medetomidine, romifidine, clonidine, brimonidine, detomidine, lofexidine, xylazine, tizanidine, guanfacine, amitraz (antihypertensives, sedatives and treatment of opiate dependence and alcohol withdrawal symptoms)
α_2B_		Kidney >> liver > brain = lung = heart = skeletal muscle (also reported in aorta and spleen)	Vasoconstriction	Antagonists: phentolamine, yohimbine, idazoxan, atipamezole, trazodone, mianserin, mirtazapine (aphrodisiac, antidepressants, reversal of α_2_-AR agonist-induced sedation)
α_2C_		Brain = kidney (also reported in spleen, aorta, heart, liver, lung, skeletal muscle)	Presynaptic inhibition of noradrenaline release	
β_1_	G_s_ (adenylate cyclase stimulation)	Brain, lung, spleen, heart, kidney, liver, muscle	Increase of cardiac output (heart rate, contractility, automaticity, conduction), renin release from juxtaglomerular cells, lipolysis in adipose tissue	Agonists Dobutamine, isoprenaline, noradrenaline (bradycardia, heart failure, cardiogenic shock) Antagonists Metoprolol, atenolol, bisoprolol, propranolol, timolol, nebivolol (cardiac arrhythmia, congestive heart failure, glaucoma, myocardial infarction, migraine prophylaxis)
β_2_		Brain, lung, lymphocytes, skin, liver, heart	Smooth muscle relaxation, striated muscle tremor, glycogenolysis, increased mass and contraction speed, increase of cardiac output, increase of acqueous humor production in eye, dilatation of arteries, glycogenolysis and gluconeogenesis in liver, insulin secretion, broncodilation	Agonists: (short-acting) salbutamol (albuterol), levosalbutamol (levalbuterol), terbutaline, pirbuterol, procaterol, metaproterenol, fenoterol, bitolterol mesylate, ritodrine, isoprenaline, (long-acting) salmeterol, formoterol, bambuterol, clenbuterol, (ultra-long-acting) indacaterol (asthma, other effects: vasodilation in muscle and liver, relaxation of uterine muscle, and release of insulin) Antagonists: butoxamine, timolol, propranolol (glaucoma, heart attacks, hypertension, migraine headache; investigational: stage fright, post-traumatic stress disorder)
β_3_		Adipose tissue, gall bladder > small intestine > stomach, prostate > left atrium > bladder (also reported in brown adipose tissue and endothelium of coronary microarteries)	Lipolysis, thermogenesis, relaxation of miometrium and colonic smooth muscle cells, vasodilatation of coronary arteries, negative cardiac inotropic effect	Agonists: amibegron (investigational: antidepressant, anxiolytic), solabegron (overactive bladder, irritable bowel syndrome) Antagonists: SR 59230A

### Adrenergic regulation of the immune response: an overview

Together with the hypothalamic-pituitary-adrenal axis, the sympathetic nervous system represents, the major pathway involved in the cross-talk between the brain and the immune system. Sympathoadrenergic fibers innervate both primary (bone marrow and thymus) and secondary (spleen and lymph nodes) lymphoid organs, where noradrenaline and adrenaline are released from nerve varicosities and or diffuse from the bloodstream to act on AR expressed on immune cells (Elenkov et al., 2000; Straub, 2004; Marino and Cosentino, 2013).

## The innate immune system

The innate immune system is usually considered as the first line of defense against invading microorganisms, however its contribution is increasingly emerging in several noninfectious diseases, including atherosclerosis (Chávez-Sánchez et al., 2014) and its ischemic complications (Courties et al., 2014), inflammatory bowel disease (Levine and Segal, 2013), systemic sclerosis (O'Reilly, 2014), multiple sclerosis, and other demyelinating disease (Mayo et al., 2012), neurodegenerative disease (Boutajangout and Wisniewski, 2013), and only as example, obesity (Lumeng, 2013) and diabetes (Lee, 2014) or that innate immunity play a role in tumor recognition (Marcus et al., 2014) and as a barrier to organ transplantation (Farrar et al., 2013), and even in psychiatric disorders (Jones and Thomsen, 2013).

The innate immune system consists of effector molecules such as complement and antibacterial peptides as well as of effector cells. **Complement** is a proteolytic cascade system comprising around 35 different soluble and membrane-bound proteins, which is crucial for defense from microbial infections and for clearance of immune complexes and injured cells (Noris and Remuzzi, 2013). **Antibacterial peptides** are cationic peptides (i.e., with positive net charge) containing 15–45 aminoacid residues, which are both potent antibiotics usually targeting bacterial membranes as well as effective modulators of the immune response (Boman, 2003). **Innate immune cells** include: granulocytes (neutrophils, eosinophils, basophils, mast cells), monocytes/macrophages, dendritic cells (DC), natural killer (NK) cells, γδ T lymphocytes, as well as the recently described innate lymphoid cells (ILC). Immune responses in the CNS are mediated by resident microglia and astrocytes, which are innate immune cells without direct counterparts in the periphery.

Noxious stimuli alert the innate immune system through **pattern recognition receptors (PRR)**, which can be activated by both exogenous pathogen-associated molecular patterns (PAMP) and endogenous danger (or damage)-associated molecular patterns (DAMP). PRR includes families of toll-like receptors (TLR, “toll” being the German word for “amazing” or “great”), NOD-like receptors (NLR, NOD standing for nucleotide-binding oligomerization domain), C-type lectin receptors (CLR, including mannose receptors and asialoglycoprotein receptors), RIG-I-like receptors (RLR, where RIG-I stands for retinoic acid-inducible gene 1), and AIM2-like receptors (ALR, AIM-2 or “absent in melanoma 2” being part of the inflammasome and contributing to the defense against microbial DNA). Formyl peptide receptors (FPR, binding N-formyl peptides derived from the degradation of either bacterial, or host cells) and scavenger receptors (binding oxidized or acetylated low-density lipoprotein) could be also included among PRR (Kawai and Akira, 2010; Saxena and Yeretssian, 2014).

## Adrenergic modulation of the innate immune system

Several excellent reviews deal with the role of sympathoadrenergic pathways in the communication between the nervous system and the immune system (Elenkov et al., 2000; Kohm and Sanders, 2001; Marino and Cosentino, 2013; Kenney and Ganta, 2014). However, most information regards adaptive immunity, and adrenergic regulation of the innate immune response is a relatively recent issue (Elenkov et al., 2000; Wrona, 2006; Flierl et al., 2008a; Marino and Cosentino, 2013). We will hereafter revise the most recent literature concerning humoral and cellular arms of the innate immune response, with specific attention to data obtained in humans and to their clinical implications.

### Complement

Although AR-mediated modulation of complement-induced innate immune response has been characterized at least in animal models (Flierl et al., 2008a), nearly no information is available regarding any direct effect of adrenergic pathways on complement activity.

*In vitro*, adrenaline and noradrenaline may enhance in monocytes the synthesis of several complement components (such as C2, C3, C4, C5, factor B, properdin, beta 1H, and C3b inactivator), possibly through the activation of α_1_-AR (Lappin and Whaley, 1982). Adrenaline has also been reported to inhibit C5-convertase generation from C3-convertase stabilized by nickel ions (Kozlov and Lebedeva, 1998), and to inhibit the covalent binding of the nascent C4b fragment of the human complement component to IgG (Kozlov et al., 2000), however the physiological relevance of such effects was not investigated. In COS-7 transfected cells, it was shown that gC1qR (i.e., gC1q receptor, gC1q binding protein, p32, p33), a multifunctional cellular protein that interacts with components of the complement, kinin, and coagulation cascades as well as with select microbial pathogens, may bind with the carboxyl-terminal cytoplasmic domain of the α_1B_-AR (Xu et al., 1999).

Early studies in rodents suggested that α-AR stimulation might result in increased secretion with saliva of a potent kallikrein-like anticomplementary factor (Wallace et al., 1976). In rats evidence has also been provided that the β-AR agonist isoprenaline reduces the clearance function of complement receptors on Kupffer cells, an effect which is sensitive to β-AR antagonism (Loegering and Commins, 1988), and that adrenaline may affect complement activity, both acutely (reduction followed by increase and then return to normal values in 2 h) and in the long term (increase 24 h after administration and peak after 2–3 days), however the specific contribution of AR subtypes remains to be established (Vasin and Kuznetsova, 1995). Nonetheless, another study in rats reported no effect of adrenaline (infused together with corticosterone and glucagon) on liver synthesis of albumin, complement component C3, and alpha 1-acid glycoprotein (Pedersen et al., 1989). Remarkably, it has been recently shown that the complement anaphylatoxin C5a may induce cell apoptosis in adrenal medulla following cecal ligation and puncture-induced sepsis in rats, as well as apoptosis of pheochromocytoma PC12 cells *in vitro*, resulting in impaired production of noradrenaline and dopamine (Flierl et al., 2008b), revealing a novel interaction between adrenergic pathways and the complement system.

Complement proteins may also affect brain adrenergic pathways. Indeed, early reports suggest that C5a might activate α-AR in the hypothalamus, possibly explaining the neuropsychiatric symptoms sometimes associated with immune complex diseases affecting the CNS (Williams et al., 1985). Actually, C5a in the hypothalamus may act presynaptically, resulting in noradrenaline release through the activation of a specific C5a/C5ai receptor (Schupf et al., 1989).

### Antibacterial peptides

Evidence regarding an interplay between adrenergic pathways and antibacterial peptides is recent and still fragmentary, nonetheless the few available information supports its relevance. For instance, it has been reported that the β-AR agonists albuterol and formoterol may increase the antimicrobial protein short palate, lung, and nasal epithelial clone 1 (SPLUNC1) (but not β-defensin-2/hBD-2) in normal and asthma airway epithelial cells, resulting in reduced *Mycoplasma pneumoniae* infection, and interleukin (IL)-13 attenuates such effects, decreasing SPLUNC1 and β-AR expression (Gross et al., 2010). A β-AR-dependent mechanism seems to be involved also in the psychological stress-induced decline in both cathelicidin and human β-defensin (hBD)-3 expression in the skin of mice. The antimicrobial peptide catestatin increased after short-term stress, but then began to decline with more sustained stress. In cultured keratinocytes, glucocorticoids downregulated catestatin expression, but β-AR blockade increased catestatin, as well as cathelicidin and hBD3 (Martin-Ezquerra et al., 2011). Human neutrophil peptides (HNP) 1–3 are expressed also in the synovial lining and adjacent sublining area (but not in deeper layers of synovial tissue) in both rheumatoid arthritis and osteoarthritis subjects, and their expression is concentration-dependently inhibited by noradrenaline (Riepl et al., 2010). Adrenergic agents may therefore enhance or inhibit the expression of antibacterial peptides, depending on the specific circumstances, however the factors contributing to the specific effects have been not yet clarified.

Antibacterial peptides may also affect the response of tissues to adrenergic stimulation. Recently, it has been reported that α-defensin-1/HNP1 has relaxing effects on adrenergic contractions of rat detrusor muscles, possibly through NF-κB pathways (Lee et al., 2011).

Finally, adrenaline and noradrenaline have been shown to downregulate *S. Typhimurium* virulence gene expression, increasing its sensitivity to the antimicrobial peptide LL-37 (Spencer et al., 2010).

### Cells

#### Granulocytes

Granulocytes (also known as polymorphonuclear leukocytes) are named after the presence of intracellular granules. Granulocytes are currently divided into different types, based on their staining characteristics: neutrophils (the most abundant, representing 40–80% of total leukocytes in normal conditions), eosinophils, basophils, and mast cells. **Neutrophils** are the major cellular arm of the innate immune system. They are phagocytic cells which are recruited to sites of infection to kill pathogens, however it is increasingly evident that they also play a key role in progression of non-infectious disease as well as in conditions characterized by chronic inflammation (Mócsai, 2013; Mayadas et al., 2014). **Eosinophils** have a prominent role in allergic diseases and inflammatory responses against helminthic parasites, and are involved in diseases involving mucosal surfaces, (e.g., allergic asthma, atopic dermatitis, and gastrointestinal disorders) (Kita, 2013). **Basophils** are similar to tissue-resident mast cells and their main function is the protection against infections with parasites, including ticks, and helminths (Karasuyama and Yamanishi, 2014). Finally, **mast cells** are tissue-resident granulocytes mainly located at the interface with the external environment. They contain granules rich in histamine and heparin, and their main roles are in allergy, wound healing, defense against pathogens, as well as in antitumor immunity (da Silva et al., 2014).

Adrenergic modulation of granulocytes has been examined mainly in neutrophil, nonetheless available evidence suggests that adrenergic agents may profoundly affect all granulocyte subtypes.

##### Neutrophils

The β-AR agonist isoprenaline inhibits the respiratory burst in human neutrophils (Nielson, 1987), an effect which has been confirmed in later studies and attributed to β_2_-AR (Brunskole Hummel et al., 2013). IL-8 production however seems to be not sensitive to adrenaline, which only slightly reduces the expression of the adhesion molecules CD15, CD44, and CD54, and only at very high concentrations (1 mM) (Wahle et al., 2005). Recently however the *in vitro* adhesion of neutrophils to endothelial cells was shown to be inhibited by adrenaline, an effect likely due to β-AR activation (Trabold et al., 2010). Desensitization of β-AR may occur after activation of the neutrophil respiratory burst (Vago et al., 1990), and neutrophils from elderly subjects may have decreased β-AR responsiveness (Cotter and O'Malley, 1983).

Ligand binding studies suggest that on average 1700–2200 β-AR are expressed on neutrophil membranes (Pohl et al., 1991; Schwab et al., 1993), while excluding the existence of α_2_-AR (Musgrave and Seifert, 1994). We have recently shown that human neutrophils express mRNA for all α- (with the only exception of α_2B_-) and β-AR, in the following order: β_3_ > β_2_ > α_1A_ > α_1B_ ~ α_2A_ ~ β_1_ = α_1D_ = α_2C_ and that exposure of cells to IL-8, a potent proinflammatory CXC-chemokine that promotes neutrophil chemotaxis and degranulation, increases mRNA levels of all AR, and that adrenaline, probably through the involvement of β-AR, profoundly affects neutrophil function (Scanzano et al., 2015).

Conditions reported to be associated with decreased β-AR expression on circulating neutrophils include: essential hypertension (Corradi et al., 1981), juvenile type I diabetes mellitus (Schwab et al., 1993), as well as strenuous physical exercise (Ratge et al., 1988; Fragala et al., 2011). Increased β-AR expression has been reported in post-traumatic stress disorder (Gurguis et al., 1999).

Circumstantial evidence suggests that neutrophils may also synthesize and release catecholamines. Noradrenaline and adrenaline (as well as dopamine and their major metabolites) have been identified human neutrophils (Cosentino et al., 1999), which may also contain some catecholamine-degrading enzymes, such as monoamine oxidase (MAO), possibly of the B type (Balsa et al., 1989). Indeed, the MAO inhibitor pargyline may lead at least *in vitro* to increased catecholamine levels in human neutrophils (Cosentino et al., 1999). Interestingly, exposure of rodent cells to lipopolysaccharide (LPS) results in catecholamine release together with induction of catecholamine-generating and degrading enzymes, and blockade of α_2_-AR or pharmacological inhibition of catecholamine synthesis may suppress (while α_2_-AR agonism or inhibition of catecholamine-degrading enzymes enhances) lung inflammation in rodent models of acute lung injury. Adrenalectomized animals show even enhanced catecholamine release from phagocytes as well as enhanced expression of catecholamine-synthesizing enzymes in these cells. Such results have been explained suggesting that in rodent phagocytes noradrenaline and adrenaline possibly activate NFκB resulting in enhanced release of tumor necrosis factor (TNF)-α, IL-1β and IL-6, and in the subsequent amplification of acute inflammation via α_2_-AR (Flierl et al., 2007, 2009). We recently identified in human neutrophils the presence mRNA for tyrosine hydroxylase, the rate-limiting enzyme in the synthesis of catecholamines, as well as for the vesicular monoamine transporter (VMAT) 2 (but not 1) (Scanzano et al., 2015).

##### Eosinophils

The role of adrenergic pathways in eosinophils has been studied mainly in relation to their role in allergic diseases and inflammation, with particular regard to the respiratory tract. Several studies indeed deal with the effects on eosinophils exerted by β_2_-AR agonists used as bronchodilators, however most of those studies do not provide clear evidence that any effects are actually due to AR-operated pathways (e.g., due to the absence of experiments with AR antagonists). This is a relevant issue, since many effects exerted by β_2_-AR agonists may actually occur through β-AR-independent mechanisms (Tachibana et al., 2002).

Early studies in humans examined the response of circulating eosinophils to noradrenaline, adrenaline, and emotional stress (Humphreys and Raab, 1950). In healthy subjects, the fall in circulating eosinophils following adrenaline injection, as well as the rise induced by the administration of β-AR antagonists is well characterized (Koch-Weser, 1968). Several decades later however β-AR detected on eosinophils, as well as on mast cells, macrophages, and neutrophils, were still considered of limited functional importance, due to rapid tachyphylaxis (Barnes, 1993).

Binding and functional data support the existence in eosinophils from patients with eosinophilia and from the peritoneal cavity of guinea pigs of β-AR possibly of the β_2_ subtype, coupled to adenylate cyclase, but apparently not involved in oxidative metabolism or degranulation (Yukawa et al., 1990). A few years later however another study showed that β_2_-AR may inhibit stimulated leukotriene C4 secretion and eosinophil peroxidase (EPO, an eosinophil granule basic protein) release in purified human peripheral blood eosinophils (Munoz et al., 1994). Further evidence for the functional relevance of β-AR in eosinophils is provided by results obtained in a model of airway inflammation induced in anesthetized rats by injecting substance P or bradykinin intravenously, where activation of β_2_-AR by the β_2_-AR agonist formoterol was shown to reduce the amount of plasma leakage and also the number of neutrophils and eosinophils that adhered to the vascular endothelium at sites of inflammation, an effect which could be antagonized by the β_2_-AR antagonist propranolol (Bowden Sulakvelidze and McDonald, 1994). Finally, EPO has been shown to decrease β-AR density on guinea pig lung membranes (Motojima et al., 1992). No information is available regarding the existence and the functional relevance on eosinophils of α-AR as well as of β_1_- or β_3_-AR.

##### Basophils

Clonidine, an α_2_-AR agonist used as antihypertensive, reduced stimulated secretion of histamine in human basophils, however the effect was inhibited by histamine receptor H2 blockers and not by α_1_-AR or α_2_-AR antagonists, thus a contribution of α_2_-AR could be ruled out (Miadonna et al., 1989). Recently, the antigenic activation of basophils isolated from the blood of atopic donors was shown to be decreased by adrenaline, an effect which was reduced by the β-AR antagonist propranolol (Mannaioni et al., 2010).

##### Mast cells

Both ligand-binding studies and functional data suggest that IgE-mediated histamine release from mast cells may be inhibited by β-AR (Masini et al., 1982). Desensitization of β-AR on human lung mast cells may occur after prolonged exposure to β-AR agonists like isoprenaline, an effect which is prevented by β-AR antagonists (Chong et al., 1995). Pharmacological evidence suggests that β-AR mediating inhibition of histamine release from human lung mast cells as well as from mast cells cultured from human peripheral blood are β_2_-AR (Chong et al., 2002; Wang and Lau, 2006). The β_2_-AR are also responsible in mast cells isolated from human intestinal mucosa for the inhibition exerted by adrenaline, noradrenaline, and salbutamol on IgE receptor-dependent release of histamine, lipid mediators, and TNF-α, as well as on proliferation, migration and adhesion to fibronectin and human endothelial cells (Gebhardt et al., 2005). Stem cell factor (SCF), the key human mast cell growth factor which primes mast cells for mediator release and markedly increases in asthmatic airways, profoundly reduces β_2_-AR-mediated inhibition of histamine release from human lung mast cells, possibly through SCF-dependent phosphorylation of Tyr350 on the β_2_-AR with immediate uncoupling of the receptor followed by receptor internalization (Cruse et al., 2010). At least one review exists about β_2_-AR in human lung mast cells, including the influence of genetic polymorphisms in the β_2_-AR gene (Kay and Peachell, 2005). A report also exists suggesting the existence of α_1_-AR in mast cells (Schulze and Fu, 1996) while recently Prey and coworkers shows that human mast cells are positive for the staining for both β_1_-AR and β_2_-AR while were negative for β_3_-AR (Prey et al., 2014).

#### Monocytes/macrophages

Between myeloid cells, monocyte/macrophages show different phenotypes, homeostatic turnover and functions in different tissues (Geissmann et al., 2010). Monocytes have long been considered as a developmental intermediate between bone marrow precursors and tissue macrophages. Monocytes and macrophages, together with DC, constitute the mononuclear phagocyte system which plays a key role maintaining tissue integrity during development and its restoration after injury, as well as the initiation and resolution of innate and adaptive immunity. Originally defined as bone marrow-derived myeloid cells circulating in the blood as monocytes and populating tissues as macrophages in the steady state and during inflammation, they have different phenotype, homeostatic turnover, and function in different tissues (Geissmann et al., 2010). Monocytes carry out specific effectors' functions during inflammation (De Kleer et al., 2014) and are usually classified in CD14++CCCD16- classical human monocytes or intermediates CD14++CCCD16+ cells.

**Monocytes** are endowed with chemokine receptors and PRR that modulate their migration from blood to tissues, where they produce proinflammatory cytokines and phagocyte cells and toxic molecules. Differentiation into DC or macrophages occurs during inflammation, and possibly in the steady state, depending on the inflammatory and PAMP/DAMP microenvironment (Serbina et al., 2008; Auffray et al., 2009).

**Macrophages** are resident phagocytic cells contributing to tissue homeostasis through clearance of apoptotic cells and production of growth factors. Macrophages are equipped with a broad range of pathogen recognition receptors that make them efficient at phagocytosis and induce production of inflammatory cytokines. Different subsets of macrophages occur in the various tissues (including liver Kupffer cells, lung alveolar, splenic and peritoneal macrophages, dermal macrophages). Microglial cells are resident macrophages in the CNS. The specific origins and functions of all these subsets however still await thorough investigation (Yona et al., 2013). Macrophages are also major players in major disease such as cardiovascular disease (Swirski and Nahrendorf, 2013), cancer (Biswas and Mantovani, 2010), diabetes (Cnop et al., 2005).

The expression of β-AR on human monocytes has been documented by both ligand binding and flow cytometric studies, which also suggested that their density may be affected by physical exercise (Ratge et al., 1988; Fragala et al., 2011). Receptor desensitization has been reported in human monocytes at least *in vitro* after prolonged β_2_-AR stimulation, possibly through up-regulation of cAMP phosphodiesterase activity (Manning et al., 1996).

The functional consequences of β-AR activation on human monocytes is usually antiinflammatory and immunosuppressive and includes: inhibition of oxygen radicals production (Schopf and Lemmel, 1983) upregulation of TNF receptors and inhibition of TNF (Guirao et al., 1997), reduction of *C. albicans* phagocytosis (Borda et al., 1998), inhibition of LPS-induced macrophage inflammatory protein-1 α (MIP-1 α) (Li et al., 2003), as well as of LPS-induced IL-18 and IL-12 production (Mizuno et al., 2005). Thus, while noradrenaline and adrenaline may have antiinflammatory effects on human monocytes, reversal of their effects may result proinflammatory. As a consequence, the use of β_2_-AR agonists has been suggested to be possibly beneficial in the treatment of sepsis through inhibiting LPS-elicited IL-18 (Mizuno et al., 2005), while recently the β_2_-AR antagonist propranolol has been shown to reduce circulating immunosuppressive M2b monocytes in severely burned children, suggesting a role for this drug in severely burned patients to reduce their susceptibility to opportunistic infections (Kobayashi et al., 2011). Adrenergic modulation of monocytes may also contribute to explain the increased risk of viral infections following highly stressful events (e.g., herpes simplex virus type-1 and varicella zoster virus), due to activation of the sympathetic nervous system. For instance, catecholamines directly stimulate the human cytomegalovirus immediate-early (IE) enhancer/promoter in monocytic cells via β_2_-AR, possibly leading to the development of an active human cytomegalovirus infection in latently infected patients (Prösch et al., 2000).

Adrenergic pathways in human monocytes may nonetheless result also in proinflammatory effects. Adrenaline may indeed increase monocyte attachment to laminin as well as oxidized-low density lipoprotein phagocytosis, two effects which both may be proinflammatory and atherogenic (Sarigianni et al., 2011). In particular, activation of β-AR have been shown, under certain conditions (e.g., in unstimulated cells), to lead to proinflammatory responses in monocytes, including: increased production of IL-18 (Takahashi et al., 2003), upregulation of IL-4-induced CD23 (low affinity IgE receptor/Fc epsilon RII) expression (Mencia-Huerta et al., 1991), and potentiated IgE/anti-IgE-induced production of IL-6 (Paul-Eugène et al., 1992, 1994), IgE (Paul-Eugène et al., 1993), increased generation of superoxide anion, nitric oxide, and TxB2 (Paul-Eugène et al., 1994). LPS- or IL-1-stimulated human monocytes exposed to β_2_-AR agonists may produce more antiinflammatory IL-10 as well as pro-inflammatory IL-8 (Kavelaars et al., 1997). Adrenaline may upregulate the surface expression of L-selectin (Rainer et al., 1999), and noradrenaline and adrenaline may increase matrix metalloproteinases (MMP)-1 in both circulating monocytes and monocyte-derived macrophages (Speidl et al., 2004). The β-AR agonist isoprenaline may decrease the response to LPS but *per se* result in increased phorbol ester-induced production of TNF-α, IL-12, and nitric oxide (Szelenyi et al., 2006). Isoprenaline may also increase LPS-induced production of IL-1β, possibly through the activation of β_1_-AR, which were directly identified by immunoblot techniques as well as by radioligand binding studies in the monocytic cell line THP-1 (Grisanti et al., 2010). Recently, β_1_-AR autoantibodies isolated from the sera of heart failure patients were shown to cause (TNF-α) secretion from the murine macrophage-like cell line RAW264.7 (Du et al., 2012).

Early pharmacological evidence suggested the occurrence of α-AR in human monocytes enhancing the synthesis of complement components (Lappin and Whaley, 1982). Culturing human circulating monocytes with dexamethasone or the β_2_-AR agonist terbutaline may indeed trigger the expression of α_1B_- and α_1D_-AR mRNA (Rouppe van der Voort et al., 1999). LPS may result in a similar effect, possibly through the activation of ERK-2 (Rouppe van der Voort et al., 2000). The proinflammatory cytokines TNF-α and IL-1β respectively upregulate and reduce α_1B_- and α_1D_-AR mRNA in the human THP-1 monocytic cell line (while IL-6 and IL-8 seem to be ineffective) (Heijnen et al., 2002). Recently, on monocytes a homogenous α_1B_-AR subtype population was identified, which changed to a heterogeneous receptor subtype expression pattern when differentiated to macrophages. The agonist phenylephrine synergistically increased LPS-induced IL-1β production and this effect was blocked in the presence of a selective α_1_-AR antagonist as well as of inhibitors of PKC, suggesting the occurrence on human monocytes of α_1_-AR mediating proinflammatory responses (Grisanti et al., 2011). Differentiation of human monocytes into macrophages may result, at least *in vitro*, in loss of β-AR responsiveness despite a functional adenylyl cyclase system (Baker and Fuller, 1995). Expression of β-AR on human macrophages is actually regulated upon activation in a stimulus-dependent manner, thus that changes in receptor number may occur in different states of cell maturation and function (Radojcic et al., 1991). Remarkably, as human monocytes adhere to surfaces and begin differentiation into macrophages, they may lose their surface β_2_-AR and hence become insensitive to the inhibitory effects of β_2_-AR agonists on LPS-induced TNF-α production, an observation which has been related to the lack of anti-inflammatory effect of β_2_-AR agonists on alveolar macrophages or in clinical asthma (Ezeamuzie et al., 2011). Activation of β_2_-AR may inhibit the production of TNF-α and of IL-6 and increase the production of IL-10 in PMA-differentiated U937 human macrophages (Izeboud et al., 1999), however care should be exerted when using these cells to study the physiopharmacology of β-AR in human monocytes/macrophages, since β_2_-AR, which are the main subtype of β-AR expressed by these cells, exhibit lower expression on undifferentiated (monocytes) than in PMA-differentiated U937 (macrophages) (Izeboud et al., 1999). Quite interestingly, it has been proposed that in severe sepsis priming via gut-derived noradrenaline may contribute to increased release in pro-inflammatory cytokines from Kupffer cells, ultimately leading to organ dysfunction. Such an effect, which has been tentatively defined “sympathetic excitotoxicity in sepsis,” could be mediated α_2_-AR on Kupffer cells (Miksa et al., 2005). Recently, in a porcine model of wound healing, both macrophage infiltration and angiogenesis were initially decreased, whereas dermal fibroblast function was impaired after treatment with β_2_-AR agonists, suggesting the potential of these drugs to improve skin scarring (Le Provost and Pullar, 2015). Interestingly, human monocyte-derived macrophages of stressed subjects displayed decreased superoxide anion-responses after stress, in direct correlation with higher plasma noradrenaline, and noradrenaline-treated cells showed reduced superoxide anion-production, an effect blocked by prior incubation with the α-AR antagonist phentolamine (Kuebler et al., 2013). Similarly, evidence about a negative effect of stress was recently shown in models of tumor in which increased levels of catecholamines contributes to the recruitment of macrophages and to the increased activation of these cells in the tumor environment (Armaiz-Pena et al., 2015). In addition negative effects were elucidated by the sympathetic activation on cancer progression. For example, metastasis in breast cancer, in mouse, was more pronounced after stress and this effects were mimicked by β2-AR stimulation (Sloan et al., 2010).

Finally, it has been reported that, at least *in vitro*, α_2_-AR stimulation of type A (macrophage-like) and B (fibroblast-like) synoviocytes produced an increase and a decrease in the respective cell number, probably through Gi-coupled PLC activation and the resulting stimulation of the PKC betaII/MAP kinase (Mishima et al., 2001), providing preliminary evidence for a role of α_2_-AR regulating local innate immunity in synovial tissues.

In summary, consistent evidence supports the occurrence of β-AR on human monocytes/macrophages: β_2_-AR are usually regarded as mainly antiinflammatory, although under certain conditions they can result in proinflammatory effects, while recent evidence suggests also the occurrence of β-AR (possibly β_1_-AR)-mediated proinflammatory responses (Grisanti et al., 2010). Alpha-AR can also occur upon appropriate stimulation and may mediate both pro- and antiinflammatory responses, but defining their functional role still requires careful investigation.

According to evidence obtained in rodent cells, monocytes/macrophages may also produce and utilize noradrenaline and adrenaline as local transmitters. Indeed, Spengler et al. (1994) showed in mouse peritoneal macrophages stimulated with LPS that the β-AR selective antagonist propranolol increased (and the α_2_-AR selective antagonist idazoxan decreased) TNF-α production. In the same study, the presence of intracellular noradrenaline was also reported and interpreted as an evidence supporting the existence of an adrenergic autocrine loop, possibly even more pronounced in macrophages obtained from rats with streptococcal-cell-wall-induced arthritis (Chou et al., 1998). Further support to the possibility that macrophages may produce endogenous catecholamines has been provided by Nguyen et al. (2011), who showed that exposure of mice to cold temperature rapidly promoted alternative activation of adipose tissue macrophages, which secreted catecholamines to induce thermogenic gene expression in brown adipose tissue and lipolysis in white adipose tissue. Absence of alternatively activated macrophages impaired metabolic adaptations to cold, whereas administration of IL-4 increased thermogenic gene expression, fatty acid mobilization, and energy expenditure, all in a macrophage-dependent manner (Nguyen et al., 2011). Finally, it should be mentioned that MAO type A is expressed in human monocytes in particular after incubation with IL-4, and upregulation of its expression may contribute in switching naive monocytes into a resolving phenotype, indirectly highlighting another potential role for endogenous adrenergic pathways in these cells (Chaitidis et al., 2004, 2005).

#### Dendritic cells

DC are specialized antigen-processing and presenting cells, with high phagocytic activity as immature cells and high cytokine producing capacity as mature cells. DC circulate in blood and migrate from tissues to lymphoid organs, regulating T cell responses both in the steady-state and during infection (Mellman and Steinman, 2001; De Kleer et al., 2014). Remarkably, DC found in the epidermis are the Langerhans cells, which derive from the bone marrow, under steady-state conditions are maintained locally, but during skin inflammation may be replaced by blood-borne progenitors (Merad et al., 2002).

Few information exists regarding adrenergic pathways in human DC. In CD40-stimulated human DC, activation of β_2_-AR increases intracellular cAMP and inhibits IL-12 production, resulting in inhibition of Th1 and promotion of Th2 differentiation (Panina-Bordignon et al., 1997). In human DC obtained from cord blood CD34+ precursor cells, noradrenaline acting through β_2_-AR and increased cAMP inhibits LPS-stimulated production of IL-23, IL-12 p40, TNF-α, and IL-6 without affecting IL-10 (Goyarts et al., 2008). This response is similar to that obtained in mouse skin DC (Maestroni, 2005, 2006), thus suggesting that noradrenaline may regulate human skin DC function resulting in decreased Th1 differentiation of CD4+ T cells. Indirect evidence for adrenergic regulation of human DC comes also from a study showing in 18 professional athletes a correlation between the increase of peripheral blood DC after intensive physical activity and serum adrenaline and noradenaline levels (as well as with the extent of heart rate elevation during exercise) (Suchánek et al., 2010). Recently, adrenaline was shown to inhibit migration of human DC through β_1_-AR signaling through arrestin2-PI3K-MMP9/CCR7 (Yang et al., 2013) and that DC functions are strongly affected by catecholamines (probably β_2_-AR mediated) that induces a profound suppression of the production of proinflammatory cytokines (Nijhuis et al., 2014).

Most of the information so far available regarding adrenergic pathways in DC has been obtained in murine DC, where AR mediate sympathetic nervous system influences on DC-T cells interactions contributing to the shaping of the appropriate adaptive immune response (reviewed by Maestroni, 2005, 2006). Both α_1_- and β_2_-AR are expressed on murine DC: α_1_-AR stimulate DC migration, which on the contrary is inhibited by β_2_-AR. Noradrenaline decreases IL-12 and increases IL-10 production in both skin and bone marrow-derived DC stimulated with bacterial TLR agonists and, as a consequence, DC-induced T helper (Th) 1 priming is impaired. Such observations may explain how reduced noradrenaline activity in the skin may promote contact sensitizers-induced Th1 responses (Maestroni, 2004). Noradrenaline also activates β_2_-AR-mediated cAMP-PKA pathways to enhance DC production of IL-33, resulting in direct Th2 differentiation and possibly contributing to the stress-related progression of Th2-associated disorders (Yanagawa et al., 2011). Sympathoadrenergic modulation of the skin innate and adaptive immune response occurring after stimulation with TLR2 (but not TLR4) agonists may promote a Th1 adaptive response possibly relevant to Th1-sustained autoimmune inflammatory skin diseases (Manni and Maestroni, 2008). In agreement with these findings, the β_2_-AR agonists salbutamol may bias DC preexposed to TLR-2 and NOD2 agonists toward increasing the Th17/Th1 cell ratio finally resulting in an IL-17 immune response, which may be relevant in defense against extracellular bacteria, in the pathogenesis of inflammatory diseases and for the antitumor response (Manni et al., 2011). Adrenaline however was also shown to lead bone marrow-derived murine DC to generate a dominant Th2/Th17 phenotype, possibly through the activation of β_2_-AR (Kim and Jones, 2010). Nonetheless, recently it was shown that β_2_-AR agonist-exposed mature murine DC displayed a reduced ability to cross-present protein antigens while retaining their exogenous peptide presentation capability, an effect which could be mediated through a nonclassical inhibitory G (Gαi/0) protein. Inhibition of cross-presentation was neither due to reduced costimulatory molecule expression nor antigen uptake, but rather to impaired phagosomal antigen degradation. A crosstalk between the TLR4 and β_2_-AR transduction pathways at the NF-κB level was also described, and *in vivo* treatment with a β_2_-AR agonist resulted in inhibition of antigen protein cross-presentation to CD8+ T cells, however with preservation of their exogenous major histocompatibility complex (MHC) class I peptide presentation capability (Hervé et al., 2013).

Recently, pharmacological evidence was also provided for the occurrence on murine DC of α_2_-AR, which may mediate enhancement of antigen capture, possibly contributing to explain immune enhancement following acute stress (Yanagawa et al., 2010). Dexmedetomidine, a highly-selective α_2_-AR agonist, has been recently shown to affect murine bone marrow-derived DC, delaying the intracellular proteolytic degradation of ovalbumin, decreasing the expression of the surface molecules I-A(b) and CD86, and suppressing Th-cell proliferation. Dexmedetomidine also suppressed DC migration, and vaccination of animals with dexmedetomidine-treated DC significantly suppressed the contact hypersensitivity reaction *in vivo* (Ueshima et al., 2013).

AR-dependent modulation of DC may be relevant also to cancer vaccine strategies. Botta and Maestroni (2008) found that β_2_-AR antagonism along with TLR2 activation at the site of intradermal cancer vaccination may either enhance the resulting antitumor response or be tolerogenic in dependence of the maturation state of the transferred DC. Manipulation of β_2_-AR expressed in the site of DC inoculation may thus profoundly influence the efficacy of the subsequent antitumor response.

#### Natural killer cells

NK cells, like other innate immune cells, were described as nonspecific in their interactions with tumors or virus-infected cells, however it is now well-defined that they express a repertoire of inhibitory receptors (some specific for MHC class I, others binding non-MHC ligands) that regulate their activation. NK cells also express activating receptors, and their complex interplay with inhibitory receptors is a matter of intense investigation. NK cells circulate through the blood, lymphatics and tissues, patrolling the body for the presence of transformed or pathogen-infected cells (Yokoyama, 2005; Lanier, 2008; Chijioke and Münz, 2013).

Human NK cells express high levels of β-AR. The highest number of β-AR was found in CD16+CD56+ NK cells, and it was even increased after physical exercise (Maisel et al., 1990). Noradrenaline and adrenaline decrease NK cell cytotoxicity through the activation of β-AR (likely β_2_-AR) (Whalen and Bankhurst, 1990; Takamoto et al., 1991), however adrenaline may also stimulate NK cell cytotoxicity at lower (submicromolar-picomolar) concentrations (Hellstrand et al., 1985). *In vitro*, β_2_-AR activation on NK cells reduces cell adhesion to endothelial cells (Benschop et al., 1994, 1997), and in human subjects administration of both adrenaline and noradrenaline modulates the migratory capacity of human NK cells via spleen-independent β_2_-AR mechanism (Schedlowski et al., 1996; Benschop et al., 1997). Nonetheless, evidence for β-AR-dependent increase of NK cytotoxicity has been obtained in rats treated with amphetamine (Glac et al., 2006) and more recently it has been shown that also repeated social disruption in mice “primes” NK cells in the spleen and lung to be more proficient in their cytolytic and anti-viral/tumor effecter functions through β-AR activation (Tarr et al., 2012). Also in light of such evidence, the possibility that the previously reported β-AR- dependent decreased NK cells function may arise from methodological issues has been the subject of an interesting commentary (Ben-Eliyahu, 2012). Nonetheless, a recent *in vitro* study which screened 1200 in-use or previously approved drugs for their biological effect on freshly isolated human peripheral blood-derived NK cells included β_2_-AR agonists among the confirmed inhibitors (Theorell et al., 2014) and that in general acute administration of catecholamines (mimicking a stress condition) *in vivo*, through the interaction with β-AR, suppress NK activity (Rosenne et al., 2014).

Human NK cells also express α-AR. In CD16+ lymphocytes, β_2_-, α_1_-, α_2_-AR (but not β_1_-AR) were identified, and infusion of adrenaline (but not noradrenaline) significantly decreased all AR on NK cells (Jetschmann et al., 1997). Recently, rat NK cells were shown to express both α_1_- and α_2_-AR. Activation of either subtypes of α-AR augmented NK cytotoxicity, α_1_-AR possibly signaling through PLC, while α_2_-AR effect through PKA (Xiao et al., 2010).

Decreased NK activity induced by activation of β-AR is presently considered among the main mechanisms responsible for cancer progression associated with stressful conditions resulting in activation of the sympathetic nervous system (Shakhar and Ben-Eliyahu, 1998; Ben-Eliyahu et al., 2000; Page and Ben-Eliyahu, 2000). Noradrenaline however may also inhibit the generation of specific antitumor cytotoxic T lymphocytes (Kalinichenko et al., 1999), and even chemical denervation may lead to tumor growth (Brenner et al., 1992), thus suggesting a complex role of the sympathetic nervous system in the regulation of antitumor immunity.

In animal models, activation of the sympathoadrenergic system through either stressful events or direct stimulation of β-AR usually leads to compromised resistance to tumor development and metastasis (Stefanski and Ben-Eliyahu, 1996; Shakhar and Ben-Eliyahu, 1998). In a mouse model of restraint stress, plasma adrenaline significantly rose immediately after the release from restraint, while NK cells were decreased in the lungs and blood but not in the spleen. Decreased number of NK cells in the lungs and blood was reversed by the β-AR antagonist propranolol, suggesting that acute stress reduces the number of intraparenchymal lung NK cells via activation of β-AR receptors (Kanemi et al., 2005). Impairment of NK activity and reduced antitumor resistance due to stress and β-AR stimulation is affected by age (Page and Ben-Eliyahu, 2000) and by gender (Page et al., 2008). Administration of type-C CpG oligodeoxynucleotides (CpG-C ODN) was shown to improve NK activity and immunocompetence, potentially reducing metastatic dissemination after enhanced sympathetic stress responses (Goldfarb et al., 2009), and it was proposed to limit postoperative immunosuppression and metastatic progression in association with pharmacological blockade of β-AR and cyclooxygenase (COX) inhibition (Goldfarb et al., 2011). Blockade of β-AR in association with COX inhibitors have been recently proposed even in patients with hematological malignancies, based on results obtained in animals and showing that endogenous adrenaline together with prostaglandins may mediate the promoting effects of stress on leukemia progression through suppression of NK activity (Inbar et al., 2011).

Epidemiological studies support the hypothesis that exposure to β_2_-AR antagonists may indeed reduce cancer progression and mortality, e.g., in melanoma (De Giorgi et al., 2011) and in breast cancer (Powe et al., 2010), although conflicting results have also been reported (Shah et al., 2011; Choi et al., 2014). Whether such effects are related to β-AR-induced suppression of NK antitumor activity and/or to other effects of adrenaline and noradrenaline on antitumor immunity and on tumor biology is still a matter of debate. In any case, well-designed randomized clinical trials are needed for several cancer types to establish the potential of AR manipulation as antitumor therapy.

#### γδ T lymphocytes

Gamma delta (γδ) T lymphocytes are unconventional T cells that, like NK cells, functionally and phenotypically belong to both the innate and the adaptive immune system and represent a connection between the two. They represent about 1–10% of circulating T cells (and even 50% at some mucosal sites), and are involved in the defense against infectious diseases as well as in the inhibition of tumor development and progression (Carding and Egan, 2002). Recently, one study compared lymphocytosis in response to an acute speech stress task, high and low intensity concentric exercise, and isoproterenol infusion at two different doses, showing that γδ T lymphocytes were mobilized in response to all three tasks in a dose-dependent manner, and that their mobilization was greater than that of CD8+ T lymphocytes and less than NK cells. The authors suggest that mobilization of γδ T lymphocytes may provide protection in the context of situations in which antigen exposure is more likely to occur (Anane et al., 2009). Another study in healthy subjects using infusion of adrenaline at physiological concentrations confirmed such results, showing that γδ T cells, together with CCR7−CD45RA+CD8+ effector T cells, CD3+CD56+ NKT-like cells, CD16+CD56dim cytotoxic NK cells, and CD14dimCD16+ proinflammatory monocytes, show a rapid and transient increase after adrenaline. The proposed mechanism is adrenaline-induced attenuation of cell attachment to endothelium and subsequent demargination and release into the circulation to provide immediate protection from invading pathogens (Dimitrov et al., 2010).

#### Microglia

The first definition of the role of microglia in CNS was provided by Pio del Rio-Hortega in 1932 who in the work entitled “Cytology and cellular pathology of the nervous system” described the role and effects of these cell population into the brain (Del Rio-Hortega, 1932). In the CNS, microglia are resident mononuclear phagocytes involved mainly in immune responses and inflammatory diseases, which originate during embryogenesis from the yolk sac and enter the CNS quite early in the life of embryos. In a more recent paper, the complex physiology of these cells was widely clarified (Kettenmann et al., 2011) and in this review we can find informations about the presence of A-DR and several other receptors. Microglial cells play an important role in managing neuronal cell death, neurogenesis, and synaptic interactions, and they contribute to T-cell activation within the CNS (Katsumoto et al., 2014).

Adrenergic pathways have never been investigated in human microglia, therefore available evidence so far regards murine models. In murine microglia, by means of microarray and immunohistochemistry, β_2_-AR and possibly β_1_-AR and α_2A_-AR have been identified (Hertz et al., 2010). β-AR activation increases the production of IL-1 β, TNF-α, and IL-6 through cAMP and cAMP-dependent protein kinase (Tomozawa et al., 1995) as well as ERK1/2 and P38 MAPK (Wang et al., 2010). Nonetheless, noradrenaline acting on β-AR may also induce IL-1ra and IL-1 type II receptor expression in enriched cultures of murine microglia, thus protecting cortical neurons against IL-1 β-induced neurotoxicity (McNamee et al., 2010), and exposure to both β_1_- and β_2_-AR agonists decreased TNF-α, IL-6 and monocyte chemoattractant protein-1 production, prevented microglia activation, reduced inflammation and exerted neuroprotective effects in LPS-treated murine hippocampal slices (Markus et al., 2010). Both noradrenaline and isoprenaline promote amyloid β peptide uptake and degradation by murine microglial cells through activation of β_2_-AR, thus providing a potential link between central noradrenergic neurotransmission and neuroinflammatory mechanisms in Alzheimer's disease (Kong et al., 2010). Preliminary evidence obtained in human microglia-like (THP-1) cells seems to confirm the antiinflammatory and neuroprotective role of noradrenaline in Alzheimer's disease pathology. In these cells, noradrenaline suppressed Aβ1-42-mediated cytotoxicity and MCP-1 secretion, while enhancing Aβ-mediated IL-1β secretion through action at β_2_-AR, and activation of cAMP/PKA pathway and CREB (Yang et al., 2012). As regards α-AR, no information exists with the exception of a recent study in a rat model of monoarthritis, where it was shown that spinal glia, as well as dorsal root ganglion primary afferent neurons, express α_2_-AR and that the α_2_-AR agonist dexmedetomidine exerted analgesic effects involving the blockade of spinal glial activation (Xu et al., 2010).

#### Astrocytes

The term astroctyes includes at least two main categories of cells that can be divided according to their morphology and anatomical localization: protoplasmic cells and fibrous cells (Sofroniew and Vinters, 2010). The first type are present in the gray matter and were the first type of astrocytes identified by means of silver impregnation. Fibrous astrocytes are localized into the white matter and are quite different in the morphology. At present we know that astrocytes are the most abundant and heterogeneous neuroglial cells, their functions including participating in the formation of the blood–brain barrier and regulation of blood flow (by releasing several molecular mediators such as prostaglandins, nitric oxide, and arachidonic acid), maintaining the ion, pH, and transmitter homeostasis of the synaptic interstitial fluid, sensing transmitter release at the synaptic cleft and possibly releasing gliotransmitters, defending the CNS from all types of insults and disease through reactive gliosis. Astrocytes may also contribute to neuroinflammation upon severe challenges by releasing pro-inflammatory molecules (e.g., TNF-α, IL-1, IL-6) and possibly by contributing to antigen presentation under autoimmune response, although this latter function needs further investigation (Kimelberg and Nedergaard, 2010; Endo et al., 2015).

The main AR expressed by human astrocytes is the β_2_-AR, which regulates glycogen metabolism, immune responses, release of neurotrophic factors, as well as astrogliosis in response to neuronal injury. Astrocytic β_2_-AR are potent regulators of astrocytic TNF-α-activated genes, including IL-6, CXCL2, CXCL3, VCAM1, and ICAM1 expression, and in rats co-administration of the β_2_-AR agonist clenbuterol and TNF-α skewed the T cell population toward a double negative phenotype and induced a shift in the myeloid brain cell population toward a neutrophilic predominance, suggesting that astrocytic β_2_-AR and their downstream signaling pathway may serve as potential targets to modulate neuroinflammatory responses (Laureys et al., 2014). Nonetheless, β-AR stimulation together with TNF-receptor triggering may also induce synergistic IL-6 expression in astrocytes, which may contribute to neurodegeneration and glioma development (Spooren et al., 2011). Downregulation of the astrocytic β_2_-AR-pathway has been proposed to contribute to several neurological conditions such as multiple sclerosis, Alzheimer's disease, human immunodeficiency virus encephalitis, stroke, and hepatic encephalopathy (Laureys et al., 2010). In particular, regarding multiple sclerosis, available evidence indicates that β_2_-AR are decreased in astrocytes of patients, both in normal-appearing white matter as well as in chronic active and inactive plaques (De Keyser et al., 1999; Zeinstra et al., 2000), and it has been proposed that in this disease astrocytes may serve as primary (facultative) antigen-presenting cells due to a failure of β_2_-AR-mediated suppression of MHC II molecules (De Keyser et al., 2003). Astrocyte β_2_-AR dysregulation however may contribute to pathogenesis and progression of multiple sclerosis also through deficient inhibition of nitric oxide and proinflammatory cytokine production and glutamate uptake, as well as through deficient glycogenolysis and production of trophic factors (De Keyser et al., 2004), and reduced perfusion of normal-appearing white matter (De Keyser et al., 2008). Astrocytes as therapeutic targets in multiple sclerosis were challenged in a proof of concept clinical study by use of fluoxetine, which activates PKA in astrocytes. PKA is physiologically activated by β_2_-AR-mediated cAMP increase and in turn suppresses coactivator class II transactivator, which regulates MHC class II molecule transcription (De Keyser et al., 2010). Direct activation of PKA could in principle bypass the functional deficiency of astrocytes, however preliminary results need to be confirmed and extended in larger, randomized studies.

No information exists regarding α-AR, with the exception of a study showing the occurrence of α_1_-AR in astrocytes from human optic nerves (Mantyh et al., 1995). Interestingly, the human U373 MG astrocytoma cell line express α_1B_-AR coupled to phosphoinositide hydrolysis and calcium mobilization, which mediate a mitogenic response to α_1_-AR-agonists (Arias-Montaño et al., 1999).

## Conclusions and perspectives

Although adrenergic pathways represent the main channel of communication between the nervous system and the immune system, their role has received more attention as regards modulation of adaptive immunity (Elenkov et al., 2000; Cosentino and Marino, 2013; Marino and Cosentino, 2013), in comparison to innate immunity.

Consistent evidence however indicates that adrenergic mechanisms play a significant role even in immune cells. In particular, in human neutrophils migration, CD11b/CD18 expression, and oxidative metabolism are inhibited possibly through β-AR, although a contribution by α_1_- and α_2_-AR cannot be discarded. Inhibitory β-AR may occur also on NK cells, which also express α-AR with undefined functional role. Monocytes express β-AR which are usually antiinflammatory, even if in certain conditions proinflammatory responses may arise. Murine DC express β-AR which modulate DC-T cells interactions, while in human DC β_2_-AR may affect Th1/2 differentiation of CD4+ T cells. β_2_-AR dysregulation in microglia and astrocytes may contribute to neuroinflammation in autoimmune and neurodegenerative disease. As a whole, the main AR expressed on innate immune cells are β (possibly β_2_)-AR, although α-AR may occur on selected cell types and under specific conditions. Further studies however are needed to define the functional significance of α-AR-mediated influence on the innate immune response.

On these basis, evaluation of β-AR agonists as potential antiinflammatory drugs is strongly warranted. Agonists of β_2_-AR are currently used as bronchodilating agents in asthma, however the relative contribution of any eventual immunomodulating activity of these drugs to their overall therapeutic effects remains to be established. In addition, their usefulness in different inflammatory conditions such as atherosclerosis, where neutrophils are emerging key players (Marino et al., 2015), should be carefully considered. On the other side, β-AR antagonists might enhance the innate immune response, and therefore their usefulness could be evaluated e.g., in the potentiation of antitumor immunity. The possible immune effects of α-AR ligands require additional investigation.

Issues awaiting clarification include AR expression and function in the various innate immune cells subtypes, as well as their effects on the humoral innate immune system (complement, antibacterial peptides). In particular, no information is yet available on innate immune cells which have been recently discovered and characterized, such as ILC (Spits et al., 2013) and myeloid-derived suppressor cells (MDSC) (Gantt et al., 2014).

Circumstantial evidence also suggests the opportunity to apply a pharmacogenetic approach to better understand adrenergic modulation of the immune response and in particular of innate immunity. For instance, individuals who were homozygous for β_2_-AR Arg16 had higher levels of specific IgE to *Ascaris lumbricoides*, higher *A. lumbricoides* egg counts, and larger wheal sizes following skin-prick testing with *A. lumbricoides* allergen (Ramsay et al., 1999), and inhibition of IgE-mediated release of histamine from human lung mast cells is more resistant to desensitization when the β_2_-AR bears mutant (gly16 and glu27) forms compared to wild-type (arg16 and gln27) forms (Chong et al., 2000).

Nonetheless, the present knowledge about the adrenergic modulation of innate immunity already supports relevant therapeutic applications, such as the use of α_2_-AR antagonists in acute lung injury (Flierl et al., 2007, 2009), as well as of β-AR antagonists to reduce the risk of opportunistic infections in severely burned patients (Kobayashi et al., 2011). Moreover, as adrenergic pathways also provide a link between stressful events and chronic inflammatory disease such as atherosclerosis (Heidt et al., 2014), investigating neuroimmune adrenergic mechanisms will likely provide several opportunities to repurpose the wide array of sympathoadrenergic agents currently used in medical therapy for various non-immune indications for novel and previously unanticipated indications.

### Conflict of interest statement

The authors declare that the research was conducted in the absence of any commercial or financial relationships that could be construed as a potential conflict of interest.

## Footnotes

^1^Adrenoceptors. Last modified on 13/06/2014. Accessed on 02/09/2014., IUPHAR database (IUPHAR-DB). Available online at: http://www.iuphar-db.org/DATABASE/FamilyMenuForward?familyId=4.

## Abbreviations

DC: Dendritic cells
NK: Natural killer cells
γδ T lymphocytes: Gamma Delta T lymphocytes
AR: Adrenoceptors
Th: T helper lymphocytes
LC: Locus coeruleus
cAMP: Cyclic adenosine monophosphate
CNS: Central nervous system
ILC: Innate lymphoid cells
PRR: Pattern recognition receptors
PAMP: Pathogen-associated molecular patterns
DAMP: Danger (or damage)-associated molecular patterns
TLR: Toll-like receptors
NLR: NOD-like receptors
CLR: C-type lectin receptors
RLR: RIG-I-like receptors
ALR: AIM2-like receptors
FPR: Formyl peptide receptors
gC1qR: gC1q receptor
SPLUNC1: Nasal epithelial clone 1
hBD: Human β-defensin
HNP: Human neutrophil peptide
MAO: Monoamine oxidase
VMAT: Vesicular monoamine transporter
EPO: Eosinophil peroxidase
TNF: Tumor necrosis factor
SCF: Stem cell factor
MIP: Macrophage inflammatory protein
LPS: Lipopolysaccharide
IL: Interleukine
IE: Immediate-early
MMP: Matrix metalloproteinases
PKC: Protein kinase C
MHC: Major histocompatibility complex
PLC: Phospholipase C
PKA: Protein kinase A
CpG-C ODN: Type-C CpG oligodeoxynucleotides
COX: Cyclooxygenase
THP-1: Human microglia-like cells
MDSC: Myeloid-derived suppressor cells.
